# Returning to work after a hand injury: Does ethnicity matter?

**DOI:** 10.1371/journal.pone.0229982

**Published:** 2020-03-10

**Authors:** Batia S. Marom, Moshe Sharabi, Rafael S. Carel, Navah Z. Ratzon

**Affiliations:** 1 Occupational Therapy Unit, Clalit Health Services, Tiberias, Israel; 2 Sociology and Anthropology Department, Max Stern Yezreel Valley College, Yezreel Valley, Israel; 3 School of Public Health, The University of Haifa, Haifa, Israel; 4 Department of Occupational Therapy, Sackler Faculty of Medicine, Tel Aviv University, Tel Aviv, Israel; University of Toronto, CANADA

## Abstract

**Background:**

Hand injuries (HI) are common and may limit participation in work. The objective of this study is to examine the effect of ethnicity and other prognostic variables on return-to-work (RTW) among male manual workers after acute HI.

**Methods:**

A cohort of 178 subjects (90 Arabs, 88 Jews) aged 22 to 65 was studied. Trained bilingual occupational therapists evaluated and interviewed the subjects, using structured validated questionnaires for evaluating personal and environmental factors, body function and structure, and activity limitation and participation restrictions. Employment status 3 months post injury was assessed by a telephone interview. To establish a predictive model for RTW, ethnicity and certain variables of the four domains mentioned above were analyzed using logistic regression analysis.

**Results:**

A significant difference in the rate of RTW between Jews and Arabs was found (45.5% for Jews, 28.9% for Arabs, p = 0.03) three months post HI. In the univariate regression analysis, ethnicity was associated with RTW (OR = 2.05; CI: 1.10–3.81) for Jews vs. Arabs. Using a multivariate analysis, only legal counseling, educational attainment, and the severity of disability were significantly associated with RTW.

**Conclusion:**

RTW three months post HI among manual workers is directly related to variables such as education and legal counseling and only indirectly related to ethnicity. Patients with a lower level of education and those who were engaged in legal counseling need special attention and close guidance in the process of RTW.

## Introduction

Over the last decades, the United States and many European countries have experienced significant demographic changes due to immigration from countries with cultural, religious, or ethnic backgrounds that differ from those of the majority population. With increasing ethnic diversity, caregivers are required to understand the impact of culture on human occupation and behavior [1]. Since ethnicity is one of the factors affecting health disparities [2], health professionals should improve their understanding of its effect on health [3]. Growing evidence, in many countries, consistently showed ethnic differences on the burden of injury. Ethnic minorities seem to be at higher risk of injury and mortality than the country majority of ethnic counterparts [4]. In addition, ethnic diversity has been found to influence employment status among various groups of individuals with disabilities [5].

Hand injuries (HI) are common and may result in a long-term physical and functional disability that limits participation in a wide range of activities, including work [6–8]. According to the literature HI account for up to 20% of all injuries and about 10% to 30% of all admissions to emergency units [8]. HI are common among males whose job involves physical labor [8]. Since these young male workers [7–8], have a long working life expectancy requiring continued use of hand function, it is essential to improve recovery rates. Additionally, those who do not return-to-work (RTW) at an early phase are at increased risk for long-term disability [8]. Difficulties in RTW often cause economic and social distress for the injured individuals, their family and society at large [6,7]. The number of non-life threatening injuries in 2007 in the USA was over 8 million cases. The direct and indirect medical expense was $186 billion, and accounted for 77% of all injuries and disease costs amounting to approximately $250 billion [9]. Thus, identifying and understanding factors that are likely to encourage or discourage RTW after an injury is essential. Studies agree that findings related to medical conditions and objective medical findings are not the only factors that can predict RTW. Current literature emphasizes the integration of medical, psychosocial, personal, and environmental variables [8]. The International Classification of Functioning, Disability and Health (ICF) is a framework that can be used to characterize the complexity of factors affecting RTW after HI. Four ICF domains include [6]: body dimension, (including both functions and structures); activities and participation dimension, (how an individual executes a task or action and is involved in life situations); and environmental and personal dimensions, which together comprise the context, of an individual’s life [10].

Investigators have begun to explore workers’ ethnicity as one of the contextual factors that may affect employment after injury[11], primarily due to current large demographic changes around the world. Most orthopedic injury studies that focused on ethnicity as a factor affecting employment did not consider HI specifically. A review of empirical studies on six disability groups, including traumatic brain injuries, amputations, chronic pain, myocardial infarction, spinal cord injuries, and severe mental illnesses, found that a key predictor of vocational outcomes was race/ethnicity[5]. According to this review, a higher rate of employment was reported for Caucasians compared to other minorities. A longitudinal study examined the difference in employment outcomes among Hispanic and Caucasian subjects with spinal cord injuries, and found that ethnicity had a significant effect on employment[11]. Additional studies considering other ethnic minorities such as African and Hispanic Americans found similar results[12–17]. For example, it was found that the odds of unemployment for people with spinal cord injuries at one year post-injury were 1.86 times greater for Hispanic than for Caucasian individuals[11]. In a study [12] that compared employment rate among Caucasian, African, and Hispanic Americans after spinal cord injury, it was found that the odds of being employed at one, five, and 10 years post-injury were 1.58, 2.55, and 3.03 times greater, respectively, for Caucasians compared to African Americans and 1.71, 1.86, and 1.71 higher, respectively, compared to Hispanic Americans. As mentioned above, only one study specifically examined ethnic differences in RTW after HI. The researchers found a significant difference (p = 0.005) in time to RTW between Dutch patients (7.6 ± 5.8 weeks) and patients of other ethnicities (20 ± 8.0 weeks) in the Netherlands [18].

Israeli society is heterogeneous and is composed of several ethnic groups that differ in terms of religion, culture, ethnicity, and language. Nevertheless, every Israeli resident, regardless of ethnicity, is entitled to comprehensive health care services including rehabilitation under the National Health Insurance law [4]. The two major ethnic groups in the country are Jews (74.8%) and Arabs (20.8%), more than 80% of whom are Muslims [2]. The employment rate of Jews with disabilities is 57.2%, compared with only 45.7% of Arabs [19].

A review of the literature discussing health and ethnicity shows that while there are studies about disabilities among various ethnic groups and several studies about their RTW after injuries, there are no specific studies about RTW of Arabs after injuries. Currently, a mass of Arabs immigrants are being integrated into the international labor market (especially in EU countries). Given this new reality it is incumbent upon us to explore and understand factors that affect RTW after injuries among this population.

The purpose of the present study is to examine the primary effect of ethnicity (Arabs and Jews) and other factors on rates of RTW three months post HI among manual workers in Israel. It is hypothesized that there would be a difference in the proportion of Israeli Arab and Jewish manual workers with HI who RTW three months post HI, after an adjustment for factors that significantly affect occupational status.

## Methods

### Study participants and procedure

This cohort study was based on data that was previously published [8]. In order to determine the sample size, randomized records of 30 Arab and 30 Jewish men, who were treated at the occupational therapy clinic between January—November 2011 regarding RTW were examined. Patients were divided into two groups: 1) RTW = RTW within three months post injury, 2) did not RTW = those patients who did not RTW after three months. Parameters that were included in the sample calculation were: size effect (67% compared to 43%), power of 80%, level of significance of 5%, and two tailed hypothesis. According to standard formulas, using SAS software, the calculated needed sample size was 71 participants in each group. An assumption of 20% dropout rate was made, thus the final sample size was 85 subjects in each group. As described previously [8], data was obtained from seven occupational therapy clinics where physical rehabilitation services are provided. These clinics are part of Clalit Health Services (CHS), the largest health maintenance organization (HMO) in Israel that provides comprehensive health care to more than 50% of the country's population. Subjects consisted of injured workers employed in manual professions (i.e., jobs involving physical skilled/unskilled work), aged 22–65 who sustained acute HI below the elbow whether during working hours or outside working time. Inclusion and exclusion criteria and a study protocol is provided in an earlier publication [8]. Participants were recruited between September 2012 and December 2013. In the initial assessment, validated questionnaires and measurement tools based on the protocols of the American Society of Hand Therapists were used. This was followed by a structured telephone interview three months later.

**Fig 1:**
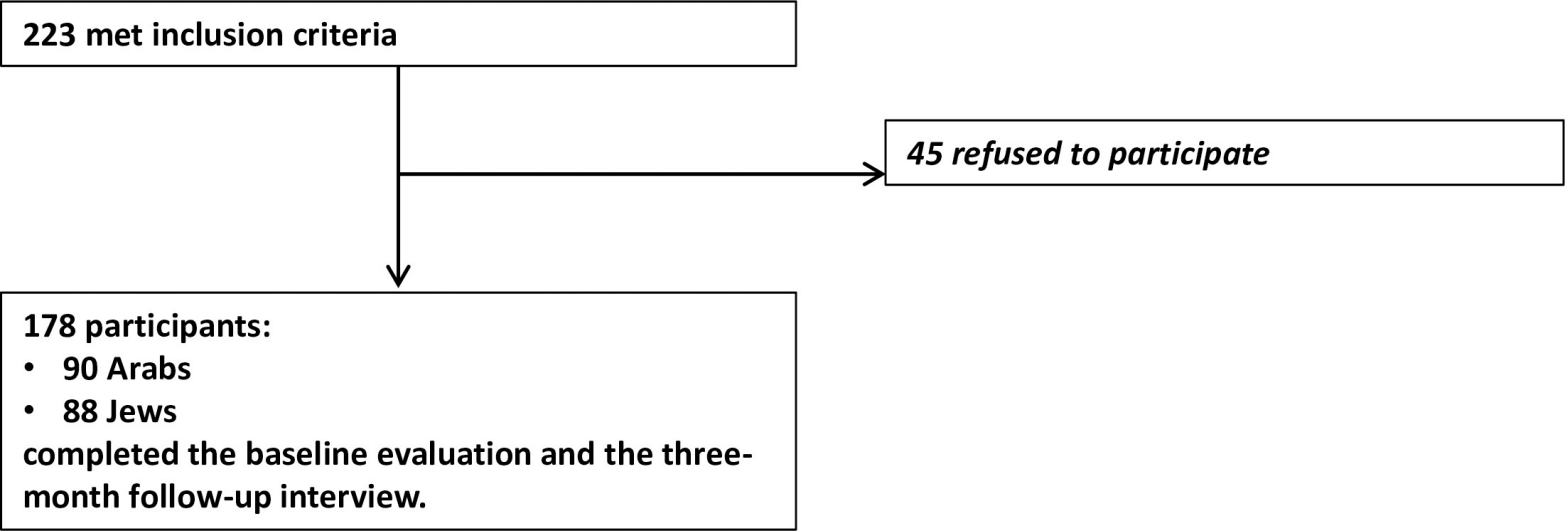
Study enrollment.

Records of 223 adult males met the inclusion criteria. However, during recruitment, 45 subjects (20.2% of the total, of whom 47% were Jews, and 53% were Arabs) refused to participate. Thus, 178 (79.8%) subjects were included in the final study group including 90 (50.6%) Arabs and 88 (49.4%) Jews (see Fig 1). All subjects completed the baseline evaluation and the three-month follow-up interview. There were no significant differences in demographic and injury characteristics between patients who refused to participate and those who entered the study.

### Ethical considerations

Ethics approval was obtained from the CHS Ethics/Helsinki Committee (057/2014). Written informed consent was provided by all persons who participated in the study.

### Measurements

The independent variables are summarized in Table 1.

**Table 1:** Description of the predictor variables for RTW with respect to four ICF components.

Independent Variable	Assessment Instrument/Data source	Scoring
• Ethnicity	Interview	Arab, Jewish
• Age	Interview	Number of years
• Education	Interview	≤12 years, >12 years
• Economic status	The updated socioeconomic scale of occupations in Israel, which uses objective criteria for level of income and education[20]	Range 0–100 (highest level)
• Marital status	Medical file	Partner, no partner
• Legal counsel	Interview	Patient working with legal counsel, no legal counsel
• Level of self-efficacy	**Perception of Self-Efficacy**	5-point Likert scale.
	Self-reported assessment (subjective),[21] includes six items regarding the capability of the person to participate in work, social activity, and activities that require mobility and general functioning	Final score = average score of all items, where a higher score = higher self-efficacy
• Hand strength required for job • Repetitive hand movement • Lifting heavy loads	**OHU**	Each item scored separately on a 5-point Likert scale, where 5 = highest requirement level
	Self-reported assessment[22] of type of work / classification according to level of hand use	
• Level of workload/job control • Level of work social support	**Occupational Stress questionnaire**	5-point Likert scale, where 5 = highest level of stress
	Self-reported assessment[23] of occupational stress based on psychosocial factors, job demands, control, support (JDC-S) model	In each section the average score for all items in the section is calculated. Two separate final scores: workload/job control, social support
	3-part questionnaire: workload, job control, social support	
• Physical capability of the hand	**GWIS**[24]	1. AROM scale from 0–30 2. Hand grip, score from 0–40 3. Dexterity, score from 0–15
	Sum of 3 scores: 1. Active range of motion (AROM), standard/universal goniometer performed according to standard protocol. Score combines 6 measured motions of the wrist and finger flexion. 2. Hand grip, standard JAMAR hydraulic hand dynamometer according to protocol recommended by the American Society of Hand Therapists. Calculated on unaffected side with adjustment for the effect of dominance. 3. Dexterity score, Jebsen–Taylor Hand Function Test, checkers subtest. Calculated on unaffected side with adjustment for the effect of dominance	
		Total GWIS sum of the scores, 0–85, where 85 = no deficit/full capability
• Pain	VAS[25]	Scale range from 0–10, where 10 = unbearable pain
• Emotional function *(extent of mental disorder resulting from a traumatic event)*	**IES**	4-point Likert scale where 4 = highest impact of the injury
	Self-reported assessment of the extent of mental disorder resulting from a traumatic event based on 15 patterns of intrusive thoughts and patterns of avoidance related to the hand injury[26]	Final scores are the average of 7 questions on intrusion and 8 questions on avoidance
• Emotional response to trauma, avoidance • Emotional response to trauma, intrusion		
• Severity of the disability	**WHODAS 2.0**	5-point Likert scale, where 5 = highest limitation
	Self-reported assessment of function and disability exploring capabilities in six domains: cognition, mobility, self-care, interpersonal interactions, life activities, participation in society, mapped onto the ICF[10,27]	Score from 0–100, where 100 indicates greater disability

The primary independent variable ethnicity was categorized according to participants’ self-report as Arabs or Jews [8]. Other socio-demographic variables that were studied included age, marital status, education, occupation, and economic status which was defined according to the objective criteria of level of income and education [20] and housing density (number of persons in the house/number of rooms). Information on legal counseling, certain occupational questionnaires, job demands, self-efficacy, the physical capability of the hand, pain, assessment of the extent of mental and psychological impacts, resulting from a traumatic event and the severity of the disability are described in greater details in a previous publication [8]. All instruments used in this study were found to be reliable and valid as described elsewhere [10, 20–27].

#### Dependent variable

The dependent variable was a categorical variable indicating whether the subject RTW (paid employment full time or part time) or did not RTW according to the interview three months after injury.

### Statistical analysis

Data were processed using SPSS (IBM SPSS Statistics for Windows, Version 20.0, Armonk, NY, USA). Descriptive statistics included frequencies, means, standard deviations, and confidence intervals. The Shapiro–Wilk test was used to check the normality of distribution. To identify potentially confounding factors, the ethnic groups were compared in relation to demographic and injury variables. The Mann–Whitney or chi-square tests were used, depending on the distribution of the variables.

**Fig 2:**
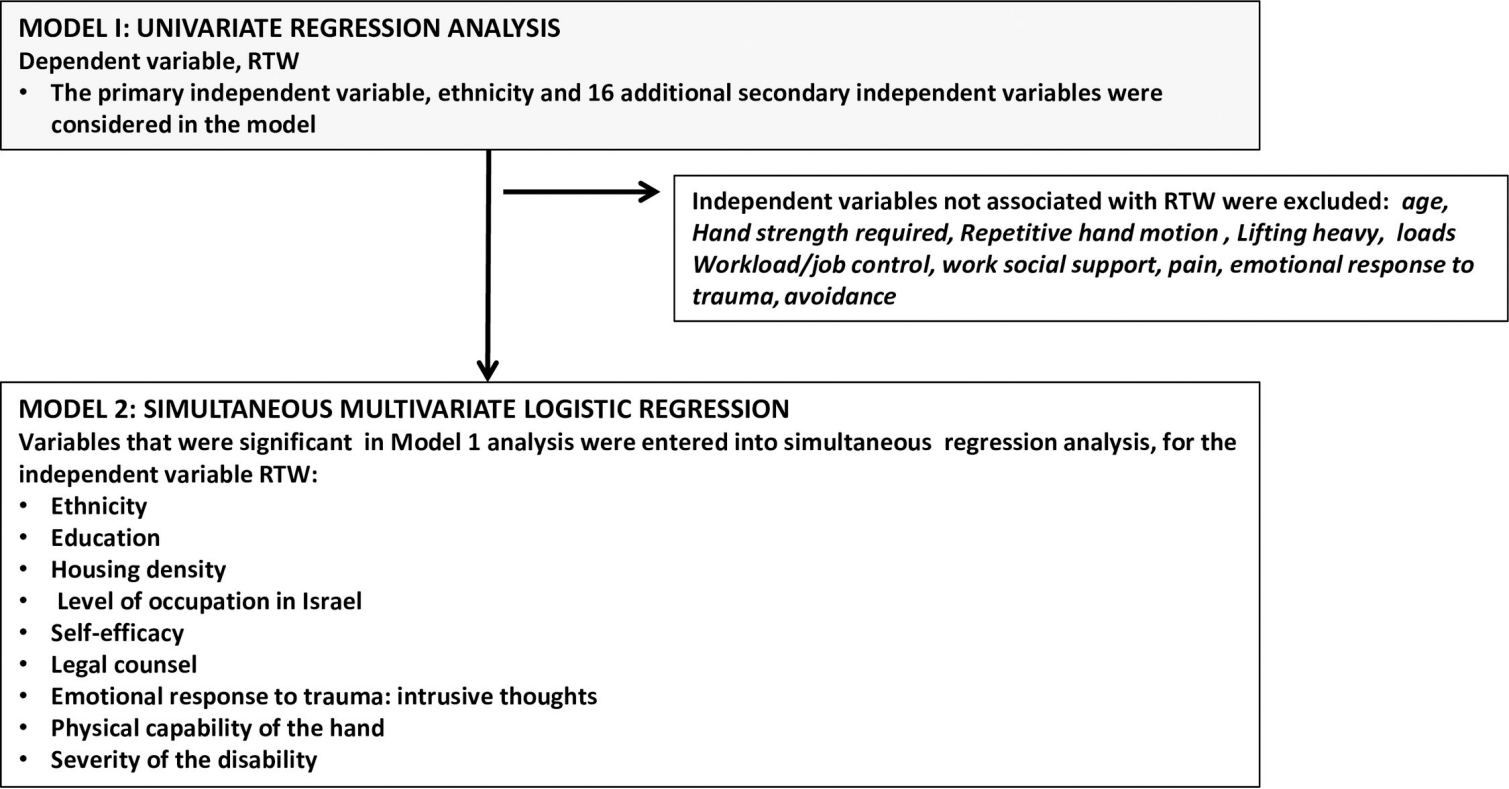
Regression analysis.

To establish a predictive model, two logistic regression analysis models were performed with the variable RTW as a dependent dichotomous variable (see Fig 2). In Model 1, the primary independent variable, ethnicity, and 16 other independent variables were examined in a simple univariate regression analysis. Only those variables that were found significant (p<0.05) in this analysis were included in the analysis in Model 2. In Model 2, simultaneous multivariate regression analysis was performed including all variables that were significant in Model 1 regression analysis (p<0.05).

## Results

### Study participants

Tables 2–4 summarize the data at the baseline for the 178 participants in the study. Overall, the Arab participants were younger and of lower socioeconomic status, their partners were less likely to work outside the home, and they had lower levels of education compared with the Jewish participants. In addition, most of the subjects in both groups worked in small businesses or in industry; 9.6% worked in a family businesses. There was a significant difference in their employment setting; 14.4% of the Arabs worked in a family business, while only 4.5% of the Jews (χ^2^(2) = 6.20, p = 0.04) did so. There was also a significant difference in the nature of their work (χ^2^(5) = 26.53, p<0.01). The majority of participants had fractures or tendon injuries (38.8% and 19.1%, respectively). Other types of injury were: soft tissue injury, amputation, injuries involving more than one compartment of the hand, and digital nerve injuries.

**Table 2:** Comparison of the sociodemographic variables for the two study groups at baseline.

**A *(Mann-Whitney)***	**Total (N = 178)**		**Jews (N = 88)**		**Arabs (N = 90)**		**Value (z)**	**CI**
	**Mean (SD)**	**Min–Max**	**Mean (SD)**	**Min-Max**	**Mean (SD)**	**Min–Max**		
Age	37.4 (11.0)	22–65.30	41.2 (11.84)	22–65.3	33.7 (8.86)	22.0–57.7	-4.27*	4.45–10.65
Housing density	1.2 (0.54)	0.25–4.0	1.07 (0.51)	0.33–4.0	1.35 (0.54)	0.25–3.0	-4.07*	-0.43–0.12
Economic status	25.76 (12.3)	7–72	28.22 (13.63)	7–72	23.37 (10.38)	8–56	-2.66*	1.25–8.44
Level of self-efficacy	3.16 (1.17)	0–5	3.32 (1.15)	0–5	3.01 (1.18)	0–5	-1.61	-0.03–0.65
**B *(chi square)***	**Total N**^†^ **(%)**		**Jews N (%)**		**Arabs N (%)**		**Value *)χ***^***2***^***)***	
Marital status No partner^‡^	57 (32.0)		24 (27.3)		33 (36.7)		1.8	
Partner	121 (68.0)		64 (72.7)		57 (63.3)			
Partner working Yes	67 (57.7)		44 (72.2)		23 (41.8)		10.89*	
No	49 (42.3)		17 (27.8)		32 (58.2)			
Education ≤12 years	135 (76.3)		61 (70.1)		74 (82.2)		**3.58**^§^	
>12 years	42 (23.7)		26 (29.9)		16 (17.8)			
Legal counsel Yes	77 (43.7)		32 (36.8)		45 (50.6)		3.39	
No	99 (56.3)		55 (63.2)		44 (49.4)			

* Sig<0.01

† Because of missing data, not all figures add up to the group total. The missing value for the variables education and legal counsel did not exceed the level of 2%. The variable partner working did not exceed the level of 5%.

‡ No partner, including 4 divorced and 2 widowed Jewish participants

§ Sig<0.05

Min–Max = minimum-maximum

Value (Z) = Mann-Whitney, Value (χ^2^) = chi square

CI = 95% confidence interval obtained from t-test

**Table 3:** Comparison of the nature of work and type of injury for members of the two groups.

	Total N (%)	Jews (%)	Arabs (%)	P
**A. Nature of work**				
Skilled workers	78 (43.8)	41 (46.6)	37 (41.1)	**<0.001**
Production, agriculture, unskilled labor	39 (21.9.)	23 (26.1)	16 (17.7)	
Construction	25 (14.0)	1 (1.1)	24 (26.7)	
Driver	21 (11.8)	15 (17.0)	6 (6.7)	
Service industry	15 (8.4)	8 (9.1)	7 (7.8)	
Total	178 (100)	88 (100)	90 (100)	
**B. Type of injury**				**>0.05**
Fracture	69 (38.8)	36 (40.9)	33 (36.7)	
Tendon Injury	34 (19.1)	20 (22.7)	14 (15.6)	
Soft tissue Injury	34 (19.1)	12 (13.6)	22 (24.4)	
Amputation	19 (10.7)	9 (10.2)	10 (11.1)	
Injury involving >1 compartment of the hand	19 (10.7)	9 (10.2)	10 (11.1)	
Digital nerve injury	3 (1.7)	2 (2.3)	1 (1.1)	
Total	178 (100)	88 (100)	90 (100)	

**Table 4:** Comparison of variables for the two groups at baseline.

	Total (N = 178)		Jews (N = 88)		Arabs (N = 90)		Z value	CI
	Mean (SD)	Min–Max	Mean (SD)	Min–Max	Mean (SD)	Min–Max		
Hand strength required for job	4.54 (0.82)	1–5	4.50(0.77)	1–5	4.59 (0.87)	1–5	-1.63	-0.33–0.15
Repetitive hand movement	4.24 (1.15)	1–5	4.23 (1.16)	1–5	4.24 (1)	1–5	-0.21	-0.36–32.0
Lifting heavy loads	3.99 (1.23)	1–5	3.86 (1.21)	1–5	4.12 (1.23)	1–5	-1.85	-6.21–0.10
Workload/job control	1.07 (0.68)	0.20–4.67	0.99 (0.60)	0.29–3.89	1.13 (0.75)	0.20–4.67	-1.69	-0.33–0.07
Level of work social support	4.14 (80)	1.50–6	4.18 (0.79)	1.50–6	4.10 (0.82)	2–5	-0.53	-0.15–0.32
**body function and structure**								
Physical capability of the hand	52.20 (15.94)	3.74–85.95	53.84 (16.03)	3.74–85.95	50.66 (15.78)	12.00–81.65	-1.29	-1.58–7.94
Pain	5.73 (2.38)	0–10	5.64 (2.20)	1–10	5.81 (2.56)	0–10	-0.94	-0.88–0.53
Emotional response to trauma, intrusion	1.82 (1.34)	0–5	1.62 (1.34)	0–5	2.01 (1.33)	0–5	-2.02*	-0.79–0.00
Emotional response to trauma, avoidance	1.48 (1.06)	0–4	1.32 (1.02)	0–3.88	1.64 (1.0)	0–4.13	-1.91*	-0.63–0.01
Severity of disability	30.21 (16.45)	0–72	28.35 (16.31)	0–72.22	32.05 (16.48)	0–69.44	-1.60	-8.57–1.15

Statistical significant difference:

*Sig<0.05, Min–Max = minimum-maximom,(Z) = Mann-Whitney, CI = 95% confidence interval (t-test)

No significant difference was found between the groups in terms of the physical capability of the hand or pain level. However, there were significant differences between the two groups in the emotional responses: avoidance and intrusion thoughts, two variables reflecting the extent of mental and psychological impacts of trauma.

### Employment status three months after HI

At the three-month follow-up interview, 37.1% of patients reported that they had returned to work after a mean of 73.1 days of disability leave (SD = 18.61 days). There was a significant difference between the groups (χ^2^(1) = 5.23, p = 0.03). Among Jews, 45.5% (40/ 88) had returned to work, while only 28.9% (26/ 90) of the Arabs had returned.

The univariate logistic regression analysis in Model 1 showed that ethnicity was associated with RTW three months after HI. The odd for Jews was 2.05 times higher compared to Arabs (Table 5). However, in contrast to our assumption, the Wald criterion demonstrated that after adjustment for other independent variables ethnicity was no longer associated with RTW (Table 5).

**Table 5:** RTW at 3 month as a function of several explanatory variables: Simple univariate regression analysis (model 1) and multivariate logistic regressions analysis (model 2).

Variable	Model 1	Model 2
	Unadjusted odds ratio (95% CI)	Adjusted odds ratio (95% CI)
Ethnicity *(primary independent variable)*		
Jews	2.05*(1.10–3.81)	1.07 (0.46–2.47)
Arabs (reference)		
Age	1.00 (0.97–1.03)	
Education		
>12 years	**3.44**^†^ (1.77–6.71)	*3.44 (1.35–8.73)
≤12 years (reference)		
Housing density	0.40*(0.20–0.80)	0.85 (0.37–1.91)
Level of occupation in Israel	1.02*(1.00–1.05)	1.00 (0.97–1.03)
Level of self-efficacy	**1.66**^†^ (1.23–2.24)	1.26 (0.83–1.93)
Legal counsel		
Yes	**0.33**^†^ (0.17–0.65)	*0.45 (0.20–1.00)
No (reference)		
Hand strength required	0.65* (0.45–0.95)	1.06 (0.58–1.92)
Repetitive hand motion	0.75* (0.58–0.98)	0.99 (0.69–1.43)
Lifting heavy loads	0.73* (0.57–0.94)	0.88 (0.60–1.30)
Workload/job control	**0.47** (0.25–0.88)	0.87 (0.37–2.00)
work social support	1.17(0.79–1.73)	
Pain	0.88(0.77–1.00)	
Physical capability of the hand	1.02*(1.00–1.04)	0.99 (0.97–1.02)
Emotional response to trauma, intrusion	**0.64**^†^ (0.49–0.82)	0.88 (0.60–1.28)
Emotional response to trauma, avoidance	0.75(0.55–1.01)	
Severity of disability	**0.95**^†^ (0.93–0.97)	^†^**0.96** (0.93–0.99)

Statistical significant difference:

*Sig≤0.05

^†^ Sig≤0.01, CI = 95% confidence interval

In Model 2 (Table 5), only the significant predictors from Model 1 were included in the analysis. A test of the full model against a constant-only model was statistically significant, indicating that the predictors included at this step, as a set, reliably distinguished between RTW or not RTW at three-month follow-up (χ^2^ = 46.70, p = 0.00, df = 9). Nagelkerke’s R^2^ of 0.33 indicated a moderately low relationship between prediction and grouping. Prediction success overall was 72% (81.3% for no RTW and 57.4% for RTW). According to this criterion, only the use of legal counsel, level of education, and severity of the disability were significant predictors (p = 0.01, p = 0.007, and p = 0.04, respectively). The EXP (B) predicted that patients who involved lawyers in getting compensation claims were less likely to RTW, those with over 12 years of education were 3.2 times more likely to RTW. Patients with more severe disabilities were less likely to RTW three months after injury.

## Discussion

This study aimed to examine ethnicity as a factor affecting RTW among Jewish and Arab manual workers in Israel three months after HI. At first glance, a significant difference in the rate of RTW between the two groups was found. In addition, simple univariate analysis showed that ethnicity was a significant factor affecting RTW. The odds for Jewish workers to RTW were 2.05 times that of Arab workers (Model 1). This finding is consistent with earlier studies that have shown ethnic disparities in employment status after various injuries [11]. However, a more extensive analysis showed that our results did not confirm our initial hypothesis. According to a simultaneous multivariate analysis (Model 2), ethnicity did not contribute significantly when the remaining independent variables were directly compared. Similar conclusions were reached in other studies [16, 17]. These complexities suggest that ethnicity alone does not function as an isolated predictor, but rather that other variables contribute and may explain the observed differences in RTW for Jews and Arabs with HI.

Although ethnicity was not a predictor of RTW in our multivariate analysis, it is important to understand the value of cultural context. The issue of culture as a factor that influences health behavior is widely recognized [28–30]. A longitudinal study conducted in Israel that examined various factors predicting RTW among more than 45,000 Israelis after traumatic injuries including extremities injuries has recently been published [31]. This study included immigrants from the Former Soviet Union, Ethiopian Immigrants, Israeli Arabs, and other Israelis. According to this study, ethnicity was associated with delayed RTW after an adjustment of other factors was made. Arabs and immigrants from Ethiopia had a greater chance for long work absences as compared to the other research groups. More specifically, it was found that the likelihood of individuals from the Arab population not RTW within a month, a year and 2 years follow up was 50%, 66% and 74% (respectively) higher than for those from the Jewish population. Discrepancies between our study and this work could be attributed to sample size and the diversity of diagnoses. Support for these results is grounded in Schwartz’s work as illustrated below.

Exploring the meaning of work within a cultural context is important, since the cultural context may shape and modify values [32]. In order to understand the meaning one attributes to work, the personal structure of values, the nature of the job the employee performs and the processes linking these various components, it is necessary to study the cultural and social context in which the individual lives and works. Regarding the cultural differences between Jews and Arabs, it was found that while Arabs in Israel are generally connected to the “conservatism” and “hierarchy” dimensions (associated with collectivism), Israeli Jews were connected to “mastery” and “affective autonomy” dimensions (associated with individualism) [32]. The cultural differences (Individualism vs. collectivism) between Jews and Arabs are reflected in their work and life values. It was found that Jews attributed higher importance to economic (good pay) and intrinsic (interest and variety) values that represent individual characteristics [33]. Furthermore, among Jews, the value of leisure (associated with individualism) is higher than among Arabs, while the importance of religion and community (associated with collectivism) are higher among Arabs than Jews [34]. Currently there are large groups of workers of different origin in many countries with different values regarding the role of work in a person’s life. Since, culture is an important aspect of occupation [28], and of perceptions of health, disability, and illness, it is important to consider these cultural differences among certain working groups in order to examine how it affects RTW. Moreover, it is critical that health care services will be aware of those cultural differences [29,30, 35].

Understanding individual cultural and ethnic uniqueness can provide a possible explanation for the lower rate of RTW among Arabs in the current study as well. One feasible explanation may relate to the differences in the role of family ties and social support between the two ethnic groups. It has been shown that Arab culture is characterized by strong family and social support [1, 34]. In the current study, Arabs were much more likely to be working in family businesses at the time of their HI than were Jews, which can compensate for economic pressures and other aspects of the needs of the injured persons. This explanation is further reinforced by the fact that, among Arabs, where spouses/partners were far less likely to be employed, the subjects had alternative sources of financial security. Similarly, the lower cost of living in the Israeli Arab society, allows for more extended unemployment and dependence on compensation [31]

Another possible cause for the different rates of RTW between the two groups is the higher level of emotional distress among Arab subjects. In Islam, there is a significance assigned to each hand [36]. According to the Muslim holy book, the Qur’an, the left hand is considered unclean and the right hand is used for eating [37]. If a person is unable to perform certain activities with the right or left hand, this can have a significant psychological impact. Other studies that examined the prevalence of mental health problems also showed that the odds for post-traumatic stress disorders (PTSD) following traumatic injuries are higher for African Americans and Hispanics as compared to non-Hispanic Caucasians counterparts. These results emphasize the importance of understanding the sociocultural mental health implications of physical trauma among different ethnic groups [3].

As depicted in Model 2’s regression analysis, the involvement of legal counseling, lower levels of education, and self-reports of greater disability were associated with lower rates of RTW. All these factors may be indirectly related to ethnicity. In general, the mean number of years of education among Arabs was significantly lower than among Jews [19]. For instance, the rate of post-secondary non-academic education among Arabs was 10.8%, versus 18.7% among Jews, and the average number of years of education among Arabs was 9.9 years, compared with 14.3 among Jews with disabilities [19]. This difference was reflected in the current study as well. Since a higher level of education is associated with more flexible employment opportunities [7, 31], a lower level of education was related to lower rates of RTW among the Arab population.

The worker compensation system in Israel is designed to provide equal access to standard medical treatment and disability reimbursement, regardless of ethnicity or socioeconomic status [38]. Yet, the fact that 50% of Arab subjects engaged a lawyer for the compensation process compared to only 36% of Jewish subjects may suggest a level of mistrust in the compensation system among Arabs. Studies have shown that sometimes Arab individuals who approach Israeli bureaucracy agencies assume that they need legal assistance 3]3].

Self-reporting of the level of disability was identified as an important factor in predicting RTW three months after HI, as reported in other studies]6]. In the current study, Arab workers reported more functional disability compared to Jewish workers. Although this difference was not significant, various studies concerning different injuries have found that minorities are more functionally disabled [13, 16], possibly suggesting that this variable is another unique barrier to RTW among Arab workers.

Our study is unique since it is one of the first studies that used the WHODAS 2.0, an instrument that is based on the ICF, for subjects after HI. Furthermore, it has important clinical relevance since it considers multiple factors related to RTW after HI, including ethnicity. Although ethnicity was not found to be a significant factor directly impacting RTW according to the final model, and as with other contextual factors, it is a fixed determining attribute of individuals and should still be considered with respect to work participation after HI, especially given the increasing rate of ethnic diversity around the world. It is important to relate to the variables that are indirectly connected to ethnicity, such as education, legal counseling, and self-report of the severity of the disability. As with ethnicity, the first two factors representing the background of an individual’s life can facilitate or hinder work function; therefore they should be considered during rehabilitation after HI. Since self-reporting of the level of disability may be a unique barrier among different ethnic groups, it is suggested that professionals treat ethnicity not just as a ‘social grouping of people,’ but as a dimension of a group with similar social, psychological, and cultural characteristics. These factors, which may affect disability, also merit attention.

## Limitations

This study has several limitations. The timing of RTW was self-reported and was not validated by other sources. Although the study was conducted with the largest health care service in northern Israel, covering almost 70% of the region, it did not include all regions and other health care services. Additionally, 20% of the patients refused to participate in this study resulting in a potential limited study population. Jews and Arabs were considered as two major ethnic groups and subgroups (Muslims, Christians, Druze, Jews from the diasporas or native Jews) were not considered. Finally, culture was determined by ethnicity; no further tools evaluated belief, behavior and other elements of culture.

## Conclusion

There was a higher rate of RTW of Jewish workers compared with Arab workers three months after HI. Factors from all ICF domains were associated with RTW. However, the study results did not show that ethnicity is the only predictor explaining the differences in the rate of RTW between Jews and Arabs individuals with HI. It is important to relate to the variables that are indirectly related to ethnicity, such as education, legal counseling, and self-reporting of the severity of the disability. In order to promote a higher rate of RTW, special attention is needed during the rehabilitation period for subjects with lower levels of education and for those who use the legal system in the compensation claims.

## Supporting information

S1 Data (XLSX)
